# First person – Georgia Cullen

**DOI:** 10.1242/bio.061706

**Published:** 2024-09-16

## Abstract

First Person is a series of interviews with the first authors of a selection of papers published in Biology Open, helping researchers promote themselves alongside their papers. Georgia Cullen is first author on ‘ Development of germline progenitors in larval queen honeybee ovaries’, published in BiO. Georgia conducted the research described in this article while a PhD student in Peter Dearden's lab at the University of Otago. She is now a post-doc in the lab of Nicholas Teets at the University of Kentucky, investigating evolution, development, germlines, soma, oogenesis, insects, and genetic modification.


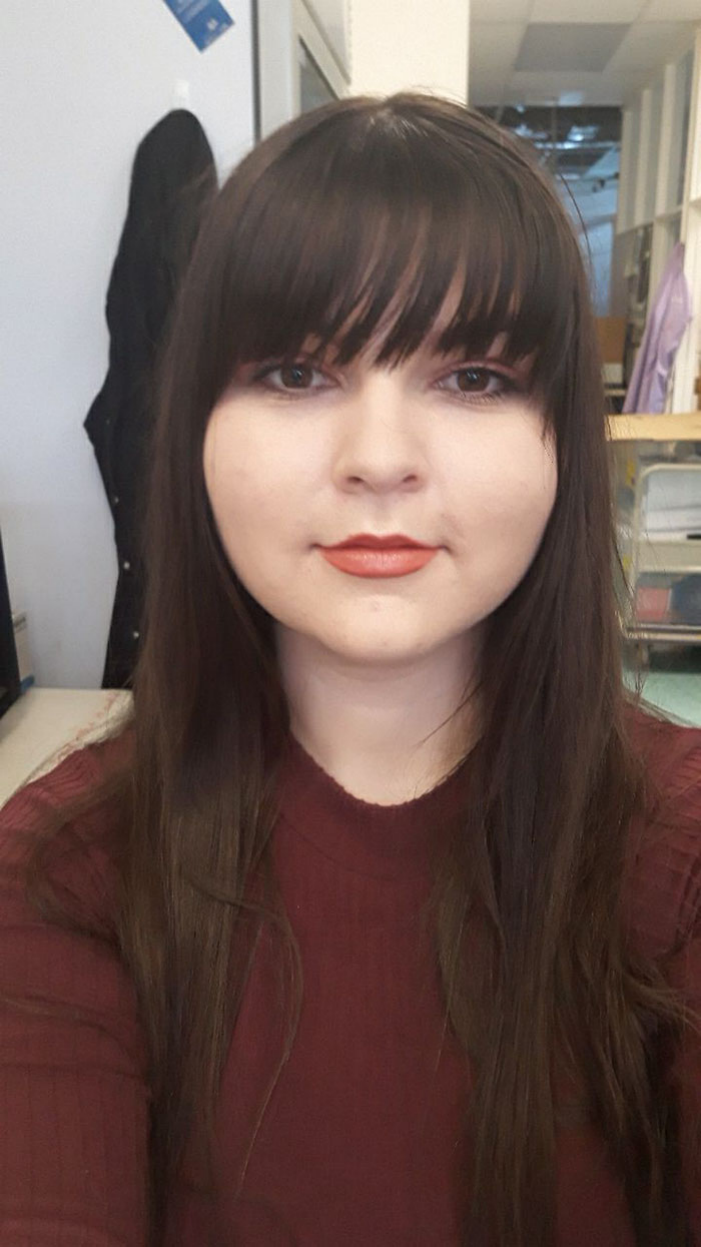


**Georgia Cullen**

**Describe your scientific journey and your current research focus**

I completed my Bachelor of Science at the University of Otago, New Zealand, majoring in zoology and genetics. During my genetics courses, I watched Peter Dearden lecture many times and was inspired to participate in small research projects in his lab. Those early investigations included RNA/DNA extraction and the optimization of *in situ* hybridization chain-reaction protocols for Kōura (New Zealand freshwater crayfish). I enjoyed working with Peter, so I applied for a Masters project in his lab, working on identifying honeybee genetic markers and investigating germline progenitors in honeybee queens. As my Masters work was successful, I wanted to explore the subject further and upgraded my Masters to a PhD. During my PhD I continued working on the honeybee germline and investigated how the germline and ovaries develop in larvae, pupae, queens and workers. After completing my PhD I wanted to learn new skills, particularly in genetic modification, thus I am now in my first Post-Doc in Nick Teets’ lab at the University of Kentucky, where I am optimizing methods of genetic modification in non-model insects.

**Who or what inspired you to become a scientist?**

I shared a love of the natural world with my grandfather, which translated into a desire to study biology. When I met and worked with Peter Dearden he inspired me to continue studying science and to develop my own research interests.

we need to consider supporting honeybee queens when they are larvae through care and feeding

**How would you explain the main finding of your paper?**

To improve honeybee populations’ survival and success, we need to consider supporting honeybee queens when they are larvae through care and feeding.

**What are the potential implications of this finding for your field of research?**

Our research identified developmental timing targets in queens. Those targets can be exploited in future experiments investigating the effects of nutrition, care or treatments on germline quality and/or quantity, giving apiarists tools to better care for their hives.

**Figure:**
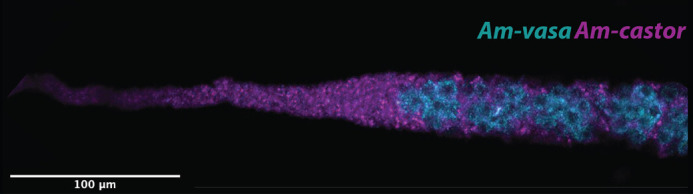
A honeybee queen ovariole showing *vasa* (germline) and *castor* (somatic) cells using *in situ* hybridization chain-reaction.

**Which part of this research project was the most rewarding?**

Presenting my results at the Congress of Genetics 2024 and receiving the Mayo Prize for best student presentation from the Genetics Society of Australasia.

**What do you enjoy most about being an early-career researcher?**

I love the moment in science when you are the only person in the world who has ever seen something.

I love the moment in science when you are the only person in the world who has ever seen something

**What piece of advice would you give to the next generation of researchers?**

You are the advocate for your own career, don't be afraid to pursue any opportunity to better yourself and your science.

**What's next for you?**

Expanding my skill set in genetic modification and diversifying my experience with as many non-model and model insects as possible.
